# Effect of final evaluation on job motivation from the perspective of nurses in Ahvaz Hospitals in 2012

**Published:** 2015

**Authors:** F Faraji Khiavi, E Amiri, S Ghobadian, R Roshankar

**Affiliations:** * Department of Health Services Administration, School of Health, Ahvaz Jundishapur University of Medical Sciences, Ahvaz, Iran; ** Rehabilitation Administration, School of Rehabilitation, Ahvaz Jundishapur University of Medical Sciences, Ahvaz, Iran

**Keywords:** job motivation, evaluation, nurses, Herzberg’s two-factor theory

## Abstract

**Background:** Increasing nurses’ motivation is among the most important and complex nursing duties. Performance evaluation system could be used as a means to improve the quantity and quality of the human resources. Therefore, current research objected to evaluate the effect of final evaluation on job motivation from the perspective of nurses in Ahvaz hospitals according to Herzberg scheme.

**Methods:** This investigation conducted in 2012. Research population included nurses in Ahvaz educational hospitals. The sample size was calculated 120 and sampling was performed based on classification and random sampling. Research instrument was a self-made questionnaire with confirmed validity through content analysis and Cronbach’s alpha calculated at 0.94. Data examined utilizing ANOVA, T-Test, and descriptive statistics.

**Results:** The nurses considered the final evaluation on management policy (3.2 ± 1.11) and monitoring (3.15 ± 1.15) among health items and responsibility (3.15 ± 1.15) and progress (3.06 ± 1.24) among motivational factors relatively effective. There was a significant association between scores of nurses' views in different age and sex groups (P = 0.01), but there was no significant association among respondents in educational level and marital status.

**Conclusion:** Experienced nurses believed that evaluation has little effect on job motivation. If annual assessment of the various job aspects are considered, managers could use it as an efficient tool to motivate nurses.

## Introduction

Hospital serves different people as a service organization with its own structure and complexity. This organization is of the main pillars of community’s health care system and its main tool is in fact the human resources [**1**]. Evaluating performance is one of the most important responsibilities of the health treatment center organizations to supply the performance quality and delivered services and the importance of this process is that it provides the opportunity to develop and acquire new knowledge and skills [**2**].

Staff performance evaluation system is considered a part of the regulatory management process as a means of growth and improvement in the quality and quantity of human resources performance. Creating incentives to improve the performance of all employees by recognizing and rewarding the hard-working staff is considered one of the main results of performance evaluation [**3**]. In fact, the main objectives of performance evaluation is to stimulate the employee’s motivation for implementation of their tasks and organization's mission [**4**]. Motivational factors include factors that will increase staff motivation and often results in an increase in the overall ability of the personnel [**5**]. Job motivation are one of the fundamental issues in human resources management and Larson and Mitchell considered it a psychological process that leads to arousal and persistence of voluntary actions of targeted people [**6**].

Nurses, as a medical team member, play an important role in improving public health [**7**]. Providing motivation to do the work and paying attention to nurses’ motivational forces are one of the most important, yet complex, management tasks in the hospital [**8**]. In all wards of the hospital, we encounter nurses who were regular, compassionate, and interested people when entering the nursing profession but usually after a few years and facing a load of problems and professional stress in the workplace, they feel tired and are even willing to leave their job. Motivation in the hospital environment is essential for providing adequate care to patients. Neglecting the motivational factors can lead to nurses’ job dissatisfaction, reducing service quality, slowing the process of recovery, and the dissatisfaction of consumers from services [**7**]. In this regard, Herzberg’s two-factor theory of “motivation-health” is one of the most comprehensive motivation theories that divides the motivating factors into two categories of motivational and health [**3**]. Health factors, including the suitability of factors such as salary, policy and administrative regulations, personal relationships with peers, supervisors and subordinates, job security, work environment and the quality of supervision can prevent dissatisfaction [but does not necessarily cause satisfaction] and the motivational factors, including factors such as the nature of work, appreciation, achievement, responsibility, growth and development cause satisfaction (but lack of them does not cause dissatisfaction) [**9**].

Researches in this field has been conducted inside and outside the country. Taghavi Larijani conducted a survey among hospitals nurses of Tehran Medical Sciences University and concluded that improving the performance of nurses using performance evaluation is the only factor associated with job motivation, compared to the other expected outcomes in the performance evaluation [**4**]. Masoud-Asl has also introduced ideal factors in the workplace, job security in the organization, policy and administrative regulations, sufficient salary, sense of responsibility at work, the feeling of being operational and adored as factors that can be effective in improving employee’s performance [**9**]. Ildez declared in his research on nurses in Turkey that the relationship with supervisors, the right to choose, working hours, and interest in the nursing profession are the motivational factors affecting the number of job resignation in nurses [**10**]. Charlsvik considered four factors in a study on nurses’ motivation, including lack of support from supervisors, having loads of responsibilities, long hours of working, and high volume tasks as the leading cause of stress among nurses [**11**]. Therefore, given that the motivation is one of the important issues that includes various aspects and despite many studies, many points have remained unknown, current research investigated the effect of final evaluation on work motivation from the perspective of nurses in Ahvaz hospitals in 2012.

## Materials and methods

This research was performed in 2012. The study population included all nurses of inpatient sectors and clinics in Jundishapur hospitals, Medical Sciences Ahvaz University, among whom 120 subjects were selected by stratified stochastic instating.

The information gathering device is a researcher-made survey with confirmed validity through content analysis. For this purpose, the questionnaire was assessed by professors and experts and required reforms were added. Cronbach’s alpha coefficient was equal to 94.4. The questionnaire involved of three sections. The initial one included 4 survey about demographic characteristics of respondents. The second part included 6 questions related to annual evaluation and staff awareness from annual evaluation. The third part included 18 items that assessed nurses’ view on the annual evaluation with different components of Herzberg’s two-factor theory (motivation-health). The points considered for each item included one point for strongly disagree, two points for disagree, three points for no idea, four points for I agree, and five points for I do not agree. Of 120 questionnaires distributed, 105 were usable. The collected data were coded and entered into SPSS software and were examined utilizing descriptive statistics, ANOVA, and T-Test.

## Results

The majority of nurses were female (86.7%), married (65.8%) and had BA degree (76.2%), and most of them were in the age group 30-40 years (48.6%). Final evaluation had significant effect on Herzberg’s motivation-health factors, according to nurses’ view and among the health factors, final evaluation had the greatest impact on monitoring (3.31 ± 1.03) and the least impact on job security (2.63 ± 1.6). And among the motivational factors knowledge (3.15 ± 1.15) had the greatest impact and appreciation (2.85 ± 1.31) was the least factor affected by final evaluation.

**Table 1:** Mean and standard deviation score nurses about the impact of evaluation results by the end of the job motivation

Herzberg’s factor	Domains	x̅ ± SD
Motivation factor	Growth	2.96 ± 1.15
	The nature of work	2.99 ± 1.22
	Responsibility	3.15 ± 1.15
	Development	3/ 06 ± 1.24
	Recognition and appreciation	2.58 ± 1.31
Health factor	Policy and Management	3.2 ± 1.11
	Supervision	3.31 ± 1.03
	Relationships	2.73 ± 1.08
	Condition	2.96 ± 1.23
	working conditions	2.97 ± 0.99
	Job security	2.63 ± 1.6
	salary	2.68 ± 1.18

According to the findings, there was no significant difference association nurses’ view regarding the education level and marital status. According to ANOVA test, among health factors “policy and management” and “working conditions” had significant association in nurses’ view in various age teams (P = 0.033), (P = 0.037). Among the motivation factors of Herzberg’s theory “growth” demine had also a significant association from the nurses’ view (P = 0.017).

**Table 2:** Domains of questionnaire among different age group

Herzberg’s factor	Domains	x̅ ± SD Age group (years)					
		<25	25-30	30-35	35-40	40-45	>45
Motivation factor	Policy and Management	3.18 ± 1.13	3.62 ± 1.11	3.33 ± 0/94	3.07 ± 1.1	2.84 ± 1.14	2 ± 1.02
	Supervision	3.31 ± 1.46	3.74 ± 0.31	29.3 ± 94.0	16.3 ± 97.0	23.3 ± 94.0	41.2 ± 73.0
	Relationships	87.2 ± 0.51	98.2 ± 27.1	69.2 ± 97.0	7.2 ± 0.21	61.2 ± 13.1	0.52 ± 64.0
	Condition*	3 ± 51.1	29.3 ± 38.1	0.83 ± 17.1	8.2 ± 16.1	53.2 ± 12.1	2.33 ± 0.81
	working conditions	5.3 ± 84.0	44.3 ± 0.51	7.2 ± 88.0	83.2 ± 97.0	76.2 ± 99.0	33.2 ± 51.0
	Job security*	87.2 ± 35.1	0.33 ± 31.1	58.2 ± 1.1	55.2 ± 0.51	15.2 ± 0.61	16.2 ± 75.0
	salary	75.2 ± 16.1	88.2 ± 42.1	29.2 ± 95.0	66.2 ± 3.1	76.2 ± 3.1	16.3 ± 33.1
Health factor	Growth	3 ± 51.1	59.3 ± 97.0	83.2 ± 0.41	66.2 ± 24.1	69.2 ± 1.1	5.2 ± 83.0
	The nature of work	3 ± 69.1	5.3 ± 0.11	0.83 ± 13.1	62.2 ± 24.1	84.2 ± 28.1	16.2 ± 98.0
	Responsibility	5.3 ± 41.1	25.3 ± 12.1	29.3 ± 12.1	0.73 ± 16.1	92.2 ± 18.1	5.2 ± 0.41
	Development	25.3 ± 66.1	48.3 ± 15.1	16.3 ± 3.1	76.2 ± 3.1	92.2 ± 18.1	16.2 ± 98.0
	Recognition and appreciation	68.2 ± 62.1	12.3 ± 31.1	83.2 ± 12.1	92.2 ± 4.1	69.2 ± 34.1	2 ± 0.41

The T-Test test showed a significant association in “working condition” and “job security” between male and female nurses ((P = 0.015), (P = 0.018)).

**Table 3:** The mean and Standard deviation questionnaire

Question	Minimum	Maximum	x̅ ± SD
Evaluation of staff in hospitals is to improve the policy.	-2	2	2.19 ± 1.150
Evaluation of the staff at the hospital will help improve the quality of management.	-2	2	2.22 ± 1.177
Evaluation of staff, supervision of staff easier.	-2	2	2.30 ± 1.161
Results of final evaluation on how effective supervisory authorities.	-2	2	2.33 ± 1.107
Evaluation results of improved relations between employees.	-2	2	-2.27 ± 1.129
Evaluation results in improved relationships between staff and the authorities.	-2	2	-2.23 ± 1.192
Evaluation results of the relationship between hospital staff and managers affected.	-2	2	-2.17 ± 1.180
Results of final evaluation is effective in improving the working conditions of employees.	-2	2	-2.04 ± 1.240
Evaluation results in the improvement of physical facilities and working conditions for the employees affected.	-2	2	-2.12 ± 1.217
The results of the final evaluation on improving occupational rating.	-2	2	2.07 ± 1.187
Evaluation results in increased job security to staff.	-2	2	-2.37 ± 1.166
Evaluation results of the annual salary and fee increases effective.	-2	2	-2.31 ± 1.187
Results of final evaluation helps individuals and organizations achieve their goals more effective.	-2	2	-2.04 ± 1.160
Evaluation results of the improvement is due to the nature of the employee.	-2	2	-2.01 ± 1.229
The results of the final evaluation will increase the sense of responsibility.	-2	2	0.215 ± 1.156
Final evaluation results to help people progress.	-2	2	2.07 ± 1.241
Evaluation results of which yields the efforts of staff to be detected.	-2	2	-2.04 ± 1.386
Results of final evaluation thank staff provides context.	-2	2	-2.25 ± 1.371

## Discussion and conclusion

On the interpretation of nurses’ view, scores more than 3 were considered effective and rates of less than 3 were considered ineffective. The nurses’ view about the effect of final evaluation on Herzberg’s theory’s motivational factors was effective with an average of more than 3 and the health factors ineffective with an average of less than 3. Nurses stated that the final evaluation had the most effect of all health and motivational factors of Herzberg’s theory on “supervision” and the lowest effect on “job security”. In prioritizing Herzberg’s theory, among motivational factors “responsibility” was considered as a factor that was most affected by the final evaluation. Excessive working hours and workload of nurses was found to lead to fatigue and reduced motivation of nurses to perform their roles [**12**].

Also “knowledge and appreciation” was the least affected factor. Aziz-zadeh et al. studied the view of faculty members on the motivational factors, which showed that provided conditions and job security had the highest scores among the external motivational factors and inherent interest in teaching and arrangement had the highest scores among the internal motivational factors [**13**]. Among the studied health factors, evaluation had the greatest impact on monitoring and the rest of the factors as follows: policy and management, working conditions, working status, relationships, and salary and job security. In Mahmoudi’s study (2007) on intensive care unit nurses stated the most important sections as follows: nature of work, motivation and appreciation, career advancement, success, and responsibility. Also the external factors in order of importance included supervision and monitoring, communication, policies governing the workplace, job security, work environment and the salary [**9**]. In Ebadi’s research (1995), nurses of Shahid Beheshti Hospitals in Tehran stated the following factors as the most important factors: the nature of responsibility, how to communicate with others, professional development, job security, salary, supervision conditions and technical monitoring, work environment, and policies governing the workplace [**14**]. Ali Abadi (2014) concluded that the importance of health factors (external) in creating job motivation was more than the motivational factors (internal) and announced “salary” as the most important factor in job motivation among the internal and external factors [**15**] that is inconsistent with the results of the present study. This difference in research results might be because of the difference in study population and objectives of the study.

From nurses’ view, final evaluation had relative impact on the job status, but male nurses believed that evaluation had little effect on job status. Female nurses believed that the final evaluation had little effect on job security, but men stated that evaluation had very little impact on job security. For both groups, evaluation was effective on policy, management, and monitoring. Mahmoudi (2007) established that the nature of work is more important for male nurses’ motivation and salary and job security are important motivational factor for female nurses [**8**]. It can be concluded that the final evaluation must be performed to assess nature of work, salary, and job security to be effective in improving the status. In Asl’s study (2010), no significant relationship was found between sex and Herzberg’s motivational-health factors [**9**]. It seems that the dissimilar results be due to the differences in the research environment because the study population and job distribution was more extensive.

Nurses younger than 25 years and 25-30 years agreed to the effect of final evaluation on motivation and other groups opposed to the effect of final evaluation on motivation. Parhizgar also stated that employees adapt to the job environment as they grow older [**16**]. Therefore, in this study, more experience nurses believed that evaluation had little effect on job motivation, because the more the nurses are placed in the workplace and face realities of the working environment, the more they mention evaluation as a ceremonial act, in which the internal and external motivation factors are not considered. More experienced nurses believed that hospital managers do not care about personnel evaluation and the impact of evaluation and this factor could act as a negative force to bring down the level of efficiency and effectiveness of human resources. Jabbari (2004) concluded in their study that faculty members of Isfahan Medical University in the age group 30-40 years had higher job motivation in salary than the age group 40 years [**5**]. Aliabadi (2004) concluded that faculty members aged 20-29 years introduced salary as the major factor in job motivation, while the age group 30-39 years considered job security, and the group older than 40 years considered work environment as the major factor [**15**], which is dissimilar to the findings of the current research. Mahmoudi (2007) further found a clear link among age and level of job motivation [**7**] that is consistent to the results of the current study.

The single nurses considered monitoring and responsibility as the most important factors in evaluation and married nurses considered supervision as the most important factor. The research findings revealed that there was no clear link among nurses’ view and their educational level. In Aliabadi’s study [2004] single faculty members expressed the nature of the work and responsibilities as the first priority, while married faculty members reported knowledge and appreciation from personnel, and the nature of the work [**15**].

Subjects with various educational degrees generally agreed on the role of evaluation on motivation. Nurses with different degrees stated supervision as the most important factor. High level of information and knowledge of nurses creates a feeling of power and freedom of action in nurses, reduces the rate of errors in nurses, and increases the quality of care [**17**]. The outcomes revealed that there is no clear link among nurses’ view and their educational level. Mahmoudi (2007) stated that education is not associated to the motivational factors [**7**]. Alaeenejad (1989) found in their researches that the educational level of nurses had a positive effect on the common view of nurses. And by increasing the level of education the view towards the nursing profession and work becomes more positive [**18**]. Since the majority of nurses in the present study had bachelor’s degree (76.2%), it seems that the reason of having no difference between views of different nurses is the same issue.
